# Topics of Public Interest

**Published:** 1913-12

**Authors:** 


### TOPICS OF PUBLIC INTEREST

Rats infected with plague bacilli have been caught in Seattle a mile from the water front.

WOOD ALCOHOL. The N. Y. Committee on the Prevention of Blindness has taken an interest in the suit of a young man against a Brooklyn brewery for permanent blindness due to wood alcohol fumes while varnishing the interior of a beer vat. Two of his companions were killed and, a year ago, one man was killed and one blinded in the same way at the same brewery. In co-operation with the State Factory Investigation Commission, a bill has been prepared for the State Legislature, requiring that every container of wood alcohol, under whatever trade name, shall be labeled in red, "Poison, Wood Alcohol. Do not use except where there is sufficient ventilation." Though the medical profession now well understands the insidious danger of wood alcohol, the laity needs protection and warning. This committee is doing a good work. We suggest that any physician who has had experience along this line communicate with the executive secretary, Miss Carolyn C. Van Blarcom, Room 67, 105 E. 22, New York.

AUTOMATIC TELEPHONE SERVICE. The Federal Company announces that no change in its rates nor limitation of number of messages will be made, except that the minimum rate will be $2.00 a month.

---

BASEMENT LIVING ROOMS are now prohibited by law in Missouri.

---

THE CHRISTIAN SCIENTIST ENROLLMENT is stated at 83,500 for North America, of which 5,000 are in Canada. The *Watchword of Truth* thanks God for this small number, but there would be more reason for thankfulness if Christian Scientists did not have so much more money and social influence than the average.

---

COLLEGIATE WORK REQUIRED FOR MEDICAL MATRICULANTS. The University of Arkansas and the Medical College of S. C. will require a year of college study next year. The total of medical schools now requiring at least one year of college study is 79, out of a total of 106. Excluding schools that give only two-year medical courses, that are about to merge or whose existence seems doomed, the total is between 90 and 95.

---

WOMAN PHYSICIAN WANTED. The Presbyterian Hospital and Dispensary at Tsinanfu, North China, needs a Christian woman physician. Adequate salary, quarters, traveling expenses, etc., are promised. Apply to Wilbert B. Smith, 600 Lexington Ave., New York City.

---

MINE SURGEON WANTED. The U. S. Civil Service Commission announces a competition by means of verified statements of education, experience, etc., submitted by December 8. The positions carry salaries ranging from $2400 to $3600.

---

NATIONAL MEDICAL LICENSE. Representative T. L. Reilly of Connecticut has introduced a bill to authorize the President to appoint a licensing board, consisting of two members each of the army, navy and public health medical services, at salaries of $4,000, to hold office for four years, and to have headquarters at Washington. Physicians, providing they are citizens and of good moral character, are to be licensed if they hold State licenses or if they present diplomas from recognized medical schools and fulfill the requirements of the A. M. A. A fee is to be charged in either case. The bill was referred to the committee on military affairs.

Note—A bill of this nature is desirable from many standpoints, but there are some criticisms that occur to us. In the first place, there might be objection to having the medical profession represented solely by officers of the government services. The officers selected from these services would more naturally be detailed for the special duty by the respective surgeons-general and should receive the pay of their rank, which ought to be specified and to be of the same degree for each service. If the federal license is to be granted merely on presentation of a State license, it might be more convenient to make a general provision covering this point. "Fulfillment of the requirements of the A. M. A." is somewhat vague. This Association requires for admission to membership, affiliation with State and County Societies, which necessarily follows license. While the A. M. A., through its officers, editorially and in various ways, has stood definitely for a high standard of medical education, it never has acted as a licensing body and has obviously never had direct control over licensing boards, and it is questionable whether it has ever formally adopted a standard of license in the sense implied in the bill and which could be used as a formal basis by the licensing board contemplated. Furthermore, in no spirit of disrespect, it may be pointed out that the A. M. A. is, originally, a private organization of physicians, comparatively recently incorporated under the laws of the State of Illinois, but, in this respect, not differing from hundreds of other private societies of various kinds. It is very questionable whether precedent and authority exist for recognizing its influence as a basis for legislation in this specific way, though there would be no objection to employing its standards as a model. And, finally, we fail to see how any bill of this nature can be passed by Congress, which will have the power to license a physician to practice in any given State, although the federal license might be applicable to a territory, to a ship sailing under the U. S. flag and to other strictly national subjects of control.

---

Prevalence of Venereal Disease in Buffalo. The Buffalo Society of Sanitary and Moral Prophylaxis (a branch of the American Federation for Sex Hygiene) has issued a pamphlet containing valuable statistics. A letter of inquiry was mailed to each of the 670 physicians of Buffalo last year. 181 replied, of whom 122 reported cases under treatment. The total reported of gonorrhœa was 648; 519 male and 129 female. A light is shed on social conditions by the fact that 407 of the men and 76 of the women were unmarried or separated; 112 men and 53 women married. For the year 1911, 2,807 cases were reported, the ratio of approximately 4 1/3 to one case under treatment, holding good in a general way for the various groups.

An estimate of the total for the city can scarcely be made. Nearly 100 of the physicians listed are not in active practice or are dead or removed. At least a hundred more are, on account of the nature of their work, not likely to see active venereal disease. On the other hand, latent and sequellar cases are not likely to be reported and many are not treated at all, or only inefficiently, while many treated by quacks, perhaps quite competently, would be omitted from statistics.

The statistics for syphilis were: Males under treatment, disease "acquired," 357; women, 108; "accidental infection," 20 of both sexes. 918 cases altogether were reported for 1911.

26 cases of venereal disease in children under 16 were also reported and 66 of the same for 1911.

47 per cent. of the physicians ascribed 83 per cent. of infections to clandestine prostitutes, 17 per cent. to professionals; 29 per cent. of physicians reported the infections from these two classes as equal; 24 per cent. reported 19.5 per cent. of cases due to clandestine and 79.5 per cent. to professional prostitutes.

Various other interesting statistics and opinions are cited.

---

### Centenarians

Mrs. Sarah Cox of Malden, Mass., and Mrs. Benjamin de St. Francois of Medford, Mass., celebrated their 101st anniversaries June 12, 1913.

Mrs. Erastus Winslow died in Charlton, Mass., shortly after her hundredth birthday in May, 1913.

Mrs. Freda Bidietizky of Philadelphia was found dead in bed, July 15, 1913, aged 109.

In the Decoration Day work-horse parade of Boston was a mare 21 years old, driven by a man 61, accompanied by his father and mother aged 101 and 103 respectively.

Dr. John C. Warbrick of Chicago contributes to the *Texas Med. Jour.* of July, 1913, a discussion of the contradictory reasons to which longevity is ascribed and records the following cases:

1. Mrs. J. D., of Marshall county, Illinois; mother of ten children, is over 106 years old. She says: "Don't worry."

2. Dr. P., 100 years old and in good health; 11 children; no pain and never worries; chops and saws wood daily; walks and takes out-door exercise daily, which he says has caused him to live to a good old age.

3. Mrs. R., 106 years old; has no need of glasses and hears as well as if young; 8 children; mental faculties clear; never was sick.

4. W. J. F., 101 years old; feels just as good as at 19; only once had any severe illness; stubborn determination to reach the century mark.

5. Mrs. W., 101 years old; the eleventh child in a family of 23; 18 grew to manhood and womanhood; healthful, normal life; most of it spent in the country, to which she attributes her advanced age. Worries and strains in early days, then peace and content later.

6. E. F. runs a farm at 100 years old; gets out of bed at 5 o'clock every morning and cares for horses and cattle before breakfast; says he would not have reached 100 had he not kept from tobacco and alcohol. Is active and healthy.

7. J. M., 107 years old; always violated rules advanced to those who desire long life; fond of pipe and smokes yet; spent most of life out doors and walked a good deal.

8. E. P., 102 years old; used tobacco all life; mother lived to 105 years; father chopped wood after 102.

9. Rev. J. C., 101 years old; never was ill; eats nothing but dry bread two days old; fresh fruit and black tea; mind agile and keen. Says, "Do good and aim high and the rest will take care of itself."

10. J. H., 93 years old; retired carpet weaver; full possession of all his mental faculties; no special illness of any kind; always went to bed in good season and rose early. Simple and regular in his habits. For years worked in a small room with no ventilation; took pork and other kinds of meat; always contented and never worried; always slept well; never had a headache; never wore glasses nor smoked.

11. Mrs. W. died at age of 96; always had good health; lived in the country all of her life and never had any fads; lived plainly and had a calm mind. Never worried about her food. Use of her mental faculties to the end.

12. J. A., farmer; lived to be 91; always worked hard and had no fads. Disposition bright and cheerful; early to bed and arose early. Followed ordinary diet and had a calm mind. Mental faculties clear to the end.

13. A. S., farmer, 90 years old; worked hard and followed the usual diet, such as tea, coffee, meat and potatoes. Early to bed and arose early; no fads; mental faculties clear to end.

14. Rev. J. M., retired minister; died at 95; in full possession of all mental faculties; lived simply and on ordinary diet.

Chewed food well and had a calm mind; early to bed and arose early. No fads of any kind.

15. R. T. A., minister; 103 years old; calm mind; no fads; followed the usual diet, smoked and took coffee all of his life.

16. D. P., 100 years old; had no fads of any kind; has lived plainly all of his life and had good health; full use of mental faculties.

17. Mrs. W., 100 years old; never had any fads; lives on ordinary diet and has good health.

18. W. N., 95 years old; never had any fads.

19. J. P., died at age of 86; never had any fads. Went to bed early and arose early. Lived plainly; had a calm mind and full use of mental faculties to the end.

Asa Goodwin of Sterrett, Ala., celebrated his 106th birthday with a barbecue at which 2000 persons were present. He is described as hale and hearty.

Note—We have, from time to time, gathered reports of persons living to 100 years or more. While the statistics may be of value for future reference, we ask the judgment of our subscribers as to whether it is worth while to continue these notes.

---

Legality of House of Delegates of A. M. A. Dr. G. Frank Lydston of Chicago has secured a verdict from the Appellate Court of Illinois reversing the decision of the Circuit Court two years ago. Dr. Lydston's contentions are supported as follows:

"1. That the elections of the A. M. A. are illegal, being held outside of the State of Illinois, in which the association was incorporated.

"2. That the majority initial vote in the association is cast by non-members.

"3. That the delegate system of the A. M. A. robs the individual members of their right to vote as members of the association.

"4. That every member of the association is entitled to a direct vote in person or by proxy.

As we understand the matter, the first contention is purely a technicality, due fundamentally to the fact that the United States is—or, perhaps, here it would be better to say *are*—not a centralized country. The A. M. A., although a national organization, is necessarily chartered by a State. Unless some general provision is made for bodies of similar nature, probably requiring a constitutional amendment, the A. M. A. must continue to be, legally, a private corporation doing business under the laws of Illinois.

The second contention refers to the method of election of Delegates by the State societies. Assuming that, as in New York, all State societies are modeled after the A. M. A., the House of Delegates of the latter is elected by the Houses of Delegates of the former. But, the State delegates are, in turn, elected by the county societies. There might be some dispute as to whether the electors, in either case, consists of a majority who are not members of the A. M. A. This significance of Dr. Simmons's plan to make all members of State and County societies ipso facto members of the A. M. A., as they are potentially, is apparent. The ruling on this point, which may be overthrown by the Supreme Court of Illinois, seems to us, also a technical victory. Granting the advisability of the general plan of Delegate government, nothing could be fairer and more in accordance with political precedent, than the present method, especially when we remember that the ultimate electorate can and is strongly urged to carry membership in the A. M. A.

With the third and fourth contentions, which are virtually one, supported by the Court, Dr. Lydston has scored a victory of the utmost importance. We have held, from the time that the first draft of the Delegate plan of control was submitted for criticism, that it went too far from the principle of a pure democracy, although our main objection to the change was on account of the obvious intention to exclude members of the Medical Society of the State of New York, who had secured membership in other ways and who did not wish either to renounce allegiance to the parent society of the A. M. A. nor to be so inconsistent as to belong to the State Association also. We felt also that the exclusion mentioned was especially undesirable since a peaceable settlement of the controversy in New York State was already under way.

One of our reasons, aside from this immediate consideration, for adhering so far as possible to the theory of a pure democracy, was that the attendance at the A. M. A. meetings had not exceeded the number practicable for a direct vote or even for the direct transaction of business, with due allowance for the fact that the actual control of any organization always drifts naturally into the hands of a small circle, best adapted to and most interested in such matters. However, with the rulings as to the popular vote, personally or by proxy, in the State of Illinois, it becomes evident that this argument falls to the ground and that the House of Delegates affords a better means of representation than a vote largely by mail, transfering the right to proxies.

Without discussing the multitude of corollary problems involved, two thoughts obtrude themselves: 1. There ought to be national provision for the chartering and management of all institutions that are essentially national in scope. 2. There ought to be a definite, permanent membership, both in the national and State Medical Societies, obtainable by all members of the profession in good standing, without undue hardship or delay or possibility of unfairness but with sufficient requirements to insure a reasonable degree of interest and attendance. We believe that the plan formerly followed by the Medical Society of the State of New York, with minor modifications, could be permanently available, since it has demonstrated its practicability for many years, on a fairly large scale.

Now, we want to say a few plain words about the general contest, of which this suit is merely an episode. Every unbiased critic must admit that, whether the means employed have suited him or not, the A. M. A. has made great progress in the last fifteen years, both in numbers, quality of scientific work and in value of service to the individual member. And we think that it must also be admitted that this improvement has been essentially due to one man. This man has been accused of most of the political, ethical and personal crimes known. It occurs to us that justice to all and, especially, to the accused, requires a fair investigation of these charges, and of the future as well as the past and present advisability of the system of control of the A. M. A. A strongly centralized authority, even a usurpation of authority beyond that ordinarily conceded by law, has been universally recognized as necessary in critical periods of states and organizations. But it must be recognized also, and the history of the world stands back of this statement, that a centralization of authority necessary for a political or economic revolution, is a source of dissatisfaction and of danger under routine conditions. The past must be justified by the past and the future must be determined impartially on its own merits. The profession cannot long postpone a full consideration of the problems suggested, and it must enter into the consideration with a full sense both of gratitude for the past accomplishment and of duty to the opinions of the present membership.

The Board of Trustees, *Jour. A. M. A.*, November 22, publish a statement regarding this matter, denying the truth of Dr. Lydston's statements. They emphasize a point which did not impress us as important, although stated by Dr. Lydston, that the verdicts were respectively against and for forcing the State's Attorney of Cook County, Ill., to "file a petition for a mandamus against the trustees of the association." The second verdict, favorable to Dr. Lydston, has been appealed to the Supreme

Court, as Dr. Lydston admitted it might be. The Board of Trustees continue: "The decision does not in any way affect the A. M. A., but relates entirely to the duties of the State's Attorney. Should the Supreme Court sustain the decision of the Appellate Court, all it would mean would be that the State's Attorney would have to bring quo warranto proceedings against the A. M. A." . . .

In a matter of this kind, we wish to be as impartial as possible. So far as we can see, Dr. Lydston has made no false statements, and while he has indulged in sarcasms and may have been unduly elated at a provisional success, it seems to us that the Trustees go too far in declaring that the statements and inferences that he had won a great decision over the A. M. A. "are without foundation in fact." Except for the point of view, the statements of the Trustees and of Dr. Lydston, agree. The ultimate question is not one of legal technicality but of a principle of representation or direct vote. This question should be decided, not by a legal battle but by referring the whole matter to the vote of the actual membership of the A. M. A.

---

OPENINGS FOR PHYSICIANS. The following postoffices in New York, which had physicians in 1912, are not represented in the list for 1913 New York State Directory: Barre Centre, Beechford, Carlisle, Durhamville, Ferndale, Grand Gorge, Grand Island, Hebron, Moriah Centre, New Hackensack, Olmsteadville, Piermont-on-Hudson, Plessis, Sharon, Shokan, Starkville, Stockbridge, Tuscarora, Wanakena, West Carthage, West Shokan.

---

ALVARENGA PRIZE ESSAY. The College of Physicians of Philadelphia will accept competitive essays up to May 1, the award to be made on July 14, amounting to about $180. Essays must be typewritten, marked by a motto and accompanied by a sealed envelope containing the name of the author, and must be written in English or accompanied by an English translation. They should be addressed to Dr. Thomas R. Neilson, Secretary, 19 S. 22d St., Philadelphia.

---

ANTITOXIN LABORATORY will be erected by the New York State Health Department at Guilderland, near Albany.

---

AWARD BY SMITHSONIAN INSTITUTION OF HODGKINS PRIZE FOR ESSAY "ON THE RELATION OF ATMOSPHERIC AIR TO TUBERCULOSIS." The prize of $1500 has been equally divided between Dr. Guy Hinsdale of Hot Springs, Virginia, for his paper on "Tuberculosis in Relation to Atmospheric Air," and Dr. S. Adolphus Knopf of New York City, for his treatise "On the Relation of Atmospheric Air to Tuberculosis."

Army Medical Corps Examinations will be held January 19, at places to be designated with regard to convenience of applicants. Applicants must be between 22 and 30, and must have had at least one year's hospital training after graduation. Twenty-six vacancies exist. Successful candidates will be commissioned first lieutenant. The salary and allowances are approximately equivalent to a gross medical income of $2,500. Full information can be secured by addressing the Surgeon General, U. S. Army, Washington, D. C.

---

Free Dispensary For Batavia. By the co-operation of various institutions, a public dispensary was opened in the Masonic Temple, November 18. A dental clinic will be maintained and a medical examiner will be present to attend to needy school children.

---

The N. Y. State Veterinary College celebrated the opening of its new hospital and clinical buildings for large and small animals, November 15. In 1868, Cornell University opened its doors, providing a Veterinary Department which was, in 1896, merged with the State College, provided for in the law of 1894. At this time there were 11 students. In 1913 there were 122 undergraduate students and a graduating class of 5.

---

Surgical Clinics of Lebanon Hospital, New York City, will be held by Dr. Parker Syms on Wednesdays at 3 P. M. The medical profession is invited to attend. Announcements will be posted on the bulletin board of the New York Academy of Medicine.

---

A Lantern Slide Lecture on Aphasia was given at the Buffalo State Hospital, November 18, by Dr. August Hoch, Director of the Psychiatric Institute of the State Hospitals.

---

Extension of City Health Association in Small Towns. Dr. Montgomery E. Leary of Rochester, at a recent meeting of rural superintendents and teachers, suggested the formation of a Monroe County Public Health Association, in affiliation with the corresponding Rochester Association, with the idea of affording pupils in rural schools the same sanitary and hygienic advantages as are now available in most large cities. As we have already pointed out, the sanitary disadvantages of condensation of population in cities have been so far counterbalanced by attention to sanitary details, that the mortality is now actually less in the average large city than in the country. To be sure, some claim that this is due to the tendency of old people to remain in or move to the country, but the actuality of this claim is by no means certain. Moreover, the city death rate is swollen by the tendency to bring serious medical and surgical cases to the city for treatment which inevitably fails to save life in many instances. Our attention was called to the necessity for greater attention to rural hygiene by a recent experience. We have always maintained a familiarity with the country, which has undoubtedly blunted our powers of observation. A guest, a typic city dweller, not used to the country, was driven through many miles of typic, fairly high-grade country. And, as we passed each farm house, he regularly remarked, "Pew," or words to that effect. Now, in and of itself, an offensive odor is not pathogenic and many distinctly rural odors are of vegetable or animal origin of quite innocent nature, but, speaking generally, a place that is surrounded by an offensive atmosphere, is unwholesome. An analysis of the sanitary factors of water supply, sewerage, disposition of garbage and other refuse, effects of proximity of domestic animals, ventilation, etc., applied to rural dwellings, schools, etc., after the analogy of municipal regulations, shows many sins of commission and omission that, save for the smaller chances for original human infection and the fact that they are not magnified by aggregation of large numbers, would result in the retribution of epidemic disease.

---

### Losses in the Balkan Wars

CASUALTIES IN BATTLES.

| | Killed. | Wounded. |
|---|---|---|
| Turks | 50,000 | 130,000 |
| Bulgars | 50,000 | 120,000 |
| Greeks | 12,000 | 45,000 |
| Serbs | 20,000 | 45,000 |
| Montegrins | 5,000 | 10,000 |
| Total | 137,000 | 350,000 |

DEATHS FROM DISEASE.

| | |
|---|---|
| Turks | 60,000 |
| Bulgars | 10,000 |
| Greeks | 5,000 |
| Serbs | 6,000 |
| Montegrins | 2,000 |
| Deaths among prisoners | 4,000 |

DEATHS AMONG NONCOMBATANTS.

Population directly affected by war (about 6,000,000) and indirectly affected (about 20,000,000) due to massacre, disease, famine and other causes attributable to the war, about 300,000.

The Rochester State Hospital was threatened with fire, November 19. 105 patients were promptly and safely transferred from the west wing, in which the fire started, but the fire was quickly controlled with small loss, so that the patients were soon returned to their original quarters.

---

Sir W. Arbuthnot Lane, Bart., the distinguished London Surgeon, and Lady Lane have been the guests of Dr. James A. MacLeod during the past week. At the invitation of Dr. Roswell Park, Sir Arbuthnot gave a clinic at the General Hospital, and there he performed the operation for which he is widely known in this country, namely, the operation for short circuiting the ileum into the sigmoid for the relief of chronic toxæmia due to static conditions in the large bowel. This operation, whether it be accompanied by the removal of the large bowel or not, has given rise to much discussion in all parts of the world, and in some parts to marked antagonism, especially so in England, where the surgeons are inclined to conservative and well established principles. It is interesting to note, however, that in the late severe illness of her Royal Highness, the Duchess of Connaught, Sir Arbuthnot was allowed by the associated surgeons on the case to perform the operation. The operation is a radical one and should be confined to cases where the indications are clearly marked out; it most certainly should only be attempted by those of wide experience in abdominal surgery. The question of the removal of the colon is a very important one, and the indications are aptly and clearly defined by Sir Arbuthnot's words, "it should be removed in addition to the short circuiting when it asks to be removed," that is, when the colon is markedly prolapsed and free; where it is extensively tied down by adhesions, due to previous operations or inflammatory attacks, it is wise not to attempt its removal but be content to perform the simpler operation of short circuiting. It is only fair to this distinguished surgeon to say that he is not indiscriminate in the performing of these radical operations and does so only after the most careful study and X-ray investigation.

---

Wood Alcohol Blindness. Cases are reported by Benoit, *La Clin. Bruxelles,* 1911, by H. H. Tyson, *Arch. of Oph.,* Sept., 1912, by Harnack, *Münch. Med. Woch.,* Sept. 3, 1912. The last emphasizes the distinction between quinine and methyl alcohol blindness, the former being due to spasm of the retinal vessels, the latter to inflammation and degeneration of the optic nerve.

